# General practitioners’ perspectives on treatment decisions and interprofessional care for women with uncomplicated urinary tract infections: An exploratory qualitative study

**DOI:** 10.1371/journal.pone.0362328

**Published:** 2026-11-11

**Authors:** Andreas Plate, Nathalie Heinz, Thivjan Thiruchelvam, Carla Meyer-Massetti, Stephen P. Jenkinson, Corinne Steinbrüchel, Daniel Drewniak, Stefan Neuner-Jehle, Oliver Senn

**Affiliations:** 1 Institute of Primary Care, University of Zurich and University Hospital Zurich, Zurich, Switzerland; 2 Institute of Biomedical Ethics and History of Medicine, University of Zurich, Zurich, Switzerland; 3 Institute of Primary Health Care (BIHAM), University of Bern, Bern, Switzerland; 4 Institute of Pharmacology PKI, University of Bern, Bern, Switzerland; 5 Senior Health Centres of the City of Zurich GFA, Zurich, Switzerland; 6 Center of Primary and Community Care, University of Luzern, Luzern, Switzerland; The University of Sydney, AUSTRALIA

## Abstract

**Background:**

Antibiotic-sparing strategies, such as delayed prescribing or symptomatic therapy, can reduce antibiotic use in uncomplicated urinary tract infections (UTIs), yet their implementation in primary care remains inconsistent. Within this context, non-physician medical staff within general practices and community pharmacies play increasingly important roles in patient counselling and care coordination, potentially shaping treatment expectations and treatment decisions. This study investigated how general practitioners (GPs) make treatment decisions in the management of uncomplicated UTIs in women and examined their views on the roles of non-physician medical staff and community pharmacies in supporting antibiotic-sparing management.

**Methods:**

We conducted an exploratory qualitative interview study with GPs who regularly manage women with uncomplicated UTIs. Virtual, semi-structured interviews were conducted. Interviews were transcribed verbatim and analyzed thematically.

**Results:**

Fifteen GPs participated; their mean age was 46.6 years, 53.3% were female, and their experience in general practice ranged from 5 to 25 years. The thematic analysis generated six themes capturing clinical, relational, practical, and interprofessional influences on UTI management. Most GPs reported prior experience with antibiotic-sparing strategies, but thresholds for antibiotic prescribing varied and were shaped by clinical factors, patient expectations, trust, and practical constraints. Practice staff already influenced UTI management, both directly through triage, testing, and counselling and indirectly through practice routines and care pathways, while GPs differed in the degree of professional autonomy they considered appropriate. Pharmacy-based care was often only partly visible to GPs, with experiences varying according to local relationships and professional roles.

**Discussion:**

The findings suggest that antibiotic-sparing UTI management extends beyond the GP prescribing decision and is shaped across the broader care pathway. Clearer professional roles, appropriate training, and feasible communication structures may support more coordinated care and antimicrobial stewardship in primary care.

## Introduction

Antibiotic-sparing strategies, such as delayed prescribing and symptomatic therapy, can substantially reduce antibiotic use in patients with uncomplicated urinary tract infections (UTIs) [1]. However, their implementation in everyday primary care remains inconsistent, and available evidence suggests that immediate antibiotic treatment remains common in routine primary care [2–4]. Therapeutic decision-making in uncomplicated UTIs is complex and shaped by a combination of clinical, contextual, interpersonal, and organizational factors, including symptom severity and duration, clinical uncertainty, patient expectations and previous treatment experiences, the clinician–patient relationship, time pressure, and the timing of consultation [5–8]. Understanding how general practitioners (GPs) navigate these influences is essential to promote antibiotic-sparing management. The present study examines these decisions in the context of acute cystitis in otherwise healthy, non-pregnant women aged 18–64 years without known functional or anatomical abnormalities of the urinary tract, a clinical presentation referred to throughout this article as uncomplicated UTI.

In primary care, however, management of uncomplicated UTIs does not necessarily occur solely within the GP–patient consultation. In general practice, non-physician medical staff may contribute to triage, telephone advice, counselling, diagnostic procedures, and follow-up, with their roles and responsibilities varying across healthcare systems. Through these activities, they may already shape patients’ expectations and subsequent treatment decisions, sometimes through informal processes that are not explicitly recognized as part of treatment decision-making. This role is becoming increasingly relevant as shortages of primary care physicians are reported in many healthcare systems, including Switzerland, driving the development of new interprofessional care models and greater task sharing within primary care [9,10]. Beyond the general practice itself, the organization of medication supply also shapes the interface with other primary care providers. A distinctive feature of Swiss primary care is physician dispensing: in many cantons, GPs may dispense medications, including antibiotics, directly from their practices. Alongside this, community pharmacies increasingly serve as accessible first-line healthcare providers and, in specific contexts, pharmacists may assess and diagnose common conditions such as uncomplicated UTIs, provide counselling and treatment, or refer patients for medical evaluation when appropriate [11–13]. These different points of contact may influence how patients are managed before, during, or after a GP consultation and may therefore shape treatment expectations, continuity of care, and antibiotic use.

Previous studies have explored factors influencing GPs’ treatment decisions for uncomplicated UTIs [5,6,14], but less is known about how these decisions are embedded within this broader interprofessional primary care environment. In particular, there is limited evidence on how GPs perceive the current and potential roles of non-physician medical staff, how they view pharmacy-based management of uncomplicated UTIs, and how collaboration and information exchange between these care settings function in routine practice. This gap is especially relevant in Switzerland, where both task sharing within general practice and the role of community pharmacies in managing common conditions are evolving.

Against this background, this exploratory study aimed to examine three interrelated aspects of uncomplicated UTI management from the perspective of GPs: (1) how GPs make treatment decisions, with particular attention to antibiotic-sparing strategies; (2) how they perceive the current and potential future roles of non-physician medical staff in general practice; and (3) how they perceive pharmacy-based management and interprofessional collaboration with community pharmacies. By examining these aspects together, the study sought to better understand how treatment decisions are situated within the broader interprofessional primary care context. Given the limited understanding of how these interprofessional and contextual factors interact in everyday practice, a qualitative approach was chosen to explore GPs’ reasoning, experiences, and perceptions in depth.

## Materials and methods

### Study design, participants and procedures

We conducted an exploratory qualitative interview study with GPs serving as teaching physicians at the Institute of Primary Care in Zurich, Switzerland. At the time of recruitment, approximately 500 GPs were registered as teaching physicians with the Institute. GPs were randomly selected from this pool and invited by email between 23/04/2025 and 15/05/2025. Randomization was performed by generating random numbers, which were then used to select GPs from a predefined list. Interested participants completed an online survey that provided study information, obtained electronic informed consent, and collected basic demographic data. Eligible participants were GPs who routinely manage patients with uncomplicated UTIs, as self-declared during the online survey.

Fifteen virtual interviews were initially scheduled, with additional interviews planned until no substantively new content relevant to the research questions emerged from subsequent interviews [15]. Before recording, participants received a brief introduction and a concise explanation of the study background and objectives. The interviews were audio recorded with participant consent and saved on a secure server. All data were processed in accordance with strict data protection standards, including secure storage, restricted access, and pseudonymisation of interview data.

### Interview guide

Interviews followed a semi-structured guide developed by the study team and iteratively refined after the first three interviews to incorporate emerging aspects and improve clarity and relevance. Core questions were aligned with the study objectives and complemented by optional targeted follow-up prompts to explore emerging topics. The guide was informed by (1) a recent scoping review on the subject [7] and (2) the research team’s expertise and practical experience. It covered three main domains: (1) decision-making in uncomplicated UTI management, (2) the role of non-physician medical staff, and (3) interprofessional collaboration with community pharmacies. Before discussing their experiences, all participants were provided with a brief standardized explanation of antibiotic-sparing strategies, including initial symptomatic management and delayed or backup antibiotic prescribing. Participants were encouraged to share their experiences and perspectives openly. The interview guide reflected a predefined conceptual framework that structured the three main interview domains (see S1 Table and S2 Table).

### Analysis

All interviews were conducted virtually using Zoom (Zoom Video Communications Inc., San Jose, CA, USA) by AP. Interviews were transcribed verbatim by TT. Data were analyzed thematically, informed by Braun and Clarke’s framework [16], supported by MAXQDA software (VERBI Software, Berlin, Germany). The analysis combined deductive and inductive elements: the predefined interview domains provided an initial analytical structure, while additional codes were developed inductively from the interview material. An initial coding framework was developed from three randomly selected interviews by AP, NH, and TT and refined through discussion. TT served as the primary coder, with subsequent coding and analysis shared between TT and AP. AP reviewed and revised the coding across the complete dataset, and the coding framework was iteratively refined during the analysis. The finalized codebook was subsequently approved and applied to all interviews. All identified codes were provided in S3 Table.

After the initial coding and organization of findings according to the broad interview domains, a subsequent interpretive analysis revisited the coded dataset to identify cross-cutting patterns of shared meaning. These patterns were developed into candidate themes and iteratively refined against the coded data, with attention to divergent and contradictory accounts.

### Research team and reflexivity

The analytic team included researchers with different professional backgrounds. AP is a physician trained in general internal medicine and infectious diseases and an experienced primary care researcher; NH has a background in nursing and health sciences; and TT was a master’s student who was trained in basic principles of qualitative research and received detailed guidance on transcription and coding. The clinical background and previous research on infections and antibiotic use in primary care of AP may have influenced interview probing and interpretation of the data. Reflexive discussions within the analytic team, drawing on these different perspectives, and consideration of divergent and contradictory accounts were used to examine and challenge initial interpretations.

### Local setting

In Switzerland, women with urinary symptoms may consult a GP or, less commonly, a gynecologist. General practices typically employ Medical Practice Assistants and Medical Practice Coordinators, while Advanced Practice Nurses are less common. Medical Practice Assistants combine administrative responsibilities with basic clinical tasks such as first contact triage and counselling, blood sampling, wound care, vaccinations, and monitoring of vital signs. Clinically focused Medical Practice Coordinators possess advanced competencies that enable delegated patient care for chronic conditions, clinical assessments, and care coordination. Advanced Practice Nurses, with advanced nursing education, often work autonomously in preventive care, chronic disease management, and patient education.

Physicians in many cantons (comparable to states or provinces in other federal systems), particularly 17 of 19 German-speaking ones, are permitted to dispense medications directly from their offices, known as self-dispensing or physician dispensing. This practice is regulated at the cantonal level under the Therapeutic Products Act [17], allowing immediate patient access to drugs during consultations, though patients retain the right to request prescriptions for external pharmacies. Medical practice assistants support dispensing by preparing prescriptions for physician approval, managing medication stock (ordering, labeling, storage), and handing out drugs to patients under supervision. They handle administrative tasks like inventory and ensure compliance without independently dispensing.

In addition, patients have the option of receiving counselling and antibiotic treatment in community pharmacies. Beyond dispensing antibiotics upon presentation of a prescription, Swiss professional and regulatory frameworks position community pharmacies as accessible points for medication education and self-care advice, including for respiratory and urinary infections that are frequently treated with antibiotics. In addition, licensed community pharmacists have the right to dispense antibiotics for uncomplicated UTI in justified and exceptional cases. This involves detailed counseling and accompanying documentation. Within clearly defined professional and regulatory frameworks, pharmacists are positioned to support appropriate antibiotic use and antimicrobial stewardship through the dispensing of antibiotics and the application of evidence-based clinical guidelines [17].

### Ethics statement and reporting standard

The Cantonal Ethics Committee of Zurich determined that this study did not fall within the scope of the Swiss Human Research Act and therefore did not require ethics committee approval. All participants received written study information and provided informed consent electronically before participation.

This manuscript was prepared in accordance with the COREQ (Consolidated Criteria for Reporting Qualitative Research) checklist. All relevant information is provided within the manuscript or in S4 File.

## Results

Between 10/06/2025 and 17/07/2025, a total of 15 interviews were conducted. After 15 interviews, no substantively new content relevant to the research questions emerged from the later interviews, and no further GPs were recruited. The mean length of the interviews was 44 minutes (range 32–54 minutes). Participating GPs had a mean age of 46.6 years and 53.3% were female. GP experience ranged from 5 to 25 years. Employment levels ranged from 50% to 100%. Selected participant and practice characteristics are summarized in Table 1.

**Table 1:** Interview participant characteristics.

Participant	1	2	3	4	5	6	7	8	9	10	11	12	13	14	15
Participant characteristics															
Sex	m	m	f	f	f	f	m	m	m	m	f	f	m	f	f
Practice location	R	C	S	S	C	S	S	C	C	C	C	C	R	R	R
GPs in practice	2	≥3	≥3	≥3	≥3	1	≥3	≥3	≥3	≥3	≥3	≥3	2	≥3	2
Practice characteristics															
Member of a practice network		x	x		x	x	x	x	x	x	x	x	x	x	x
Care of walk-in patients without registration / unregistered patients				x	x				x	x					x
Care of patients at off-peak times / weekends	x	x		x	x				x	x				x	x
Direct medication dispensing in the practice	x	x	x		x	x		x	x	x		x	x	x	x
Pharmacies nearby that offer UTI consultations	x	x	x	x	x	x	x	dk	x	x	x	dk	x	dk	x
Medical Practice Coordinator – Clinical Direction		x	x	x	x		x			x		x	x		
Medical Practice Coordinator – Administrative Direction				x	x		x	x		x					x
Nurse Practitioner or Advanced Practice Nurses				x						x					
Estimated number of patients with uncomplicated UTI per month	20	8	6	20	2	4	3	20	10	20	4	6	10	4	40
Estimated proportion of UTI patients treated primarily without antibiotics (%)															
<20						x								x	
20-40	x	x	x							x		x			x
41-60				x	x		x	x	x						
61-80											x		x		
>80															
Interview information															
Interview length (minutes)	35	32	34	45	48	45	54	50	38	50	43	47	40	47	46

M: male; f: female; GP: general practitioner; UTI: Urinary tract infection; dk: don’t know. R: rural area, C: City, S: suburban area

The thematic analysis generated six themes that captured recurring patterns in GPs’ accounts of treatment decisions and interprofessional care for uncomplicated UTIs. The themes addressed clinical thresholds for antibiotic prescribing, patient relationships and expectations, practical constraints, the role of practice staff, views on professional autonomy, and experiences of pharmacy-based care. An overview of the themes and their central organizing concepts is provided in Table 2.

**Table 2:** Overview of themes identified in the thematic analysis.

Theme	Central organising concept
Antibiotic-sparing intentions were conditional, with different thresholds for prescribing	GPs commonly described antibiotic-sparing management as an initial intention, but differed in when clinical findings, symptoms, or previous treatment led them to consider antibiotics appropriate.
Trust and established expectations could support or constrain antibiotic-sparing care	Familiarity and trust could facilitate non-antibiotic management, while established expectations could also increase pressure to prescribe.
Practical constraints shaped both immediate and delayed prescribing decisions	Time pressure, access to follow-up, and timing of consultations influenced both immediate decisions and contingency planning.
Practice staff shaped care through both formal routines and informal discretion	Practice staff contributed to triage, testing, counselling, and treatment pathways through differently formalized arrangements.
Support for role expansion did not imply agreement on professional autonomy	GPs differed in how much clinical responsibility they considered appropriate for practice staff, particularly regarding antibiotic treatment.
Partial visibility shaped how GPs judged pharmacy-based care	Limited insight into pharmacy-based UTI care meant that GPs’ views were formed largely through the cases visible to them, local professional relationships, and their perceptions of pharmacists’ clinical roles and boundaries.

The table summarizes the six themes developed from the interview data and their central organizing concepts. Illustrative quotations are presented in the Results section, while a more detailed overview of individual factors influencing treatment decisions is provided in Supplemental S5 Table.

### Theme 1: Antibiotic-sparing intentions were conditional, with different thresholds for prescribing

Most GPs reported prior experience with antibiotic-sparing strategies and described using symptomatic management, with or without delayed or backup antibiotic prescribing.

*“By now, the antibiotic-sparing approach is actually the approach of choice in our practice, the approach that we largely follow, and the approach that we try to convey to our younger colleagues in teaching. However, it is still often the case that aspiration and reality are very far apart.” (GP10, Pos. 3)*

Most stated that they aimed to use an antibiotic-sparing approach initially, although their thresholds for prescribing antibiotics differed. Several GPs noted that women often presented only after having already attempted self-treatment, sometimes for several days.

*“[…] Because the patients themselves, as I experience it, actually don’t like taking antibiotics and often already treat themselves, for example with mannose. They often only come after having treated themselves for five days and it hasn’t gotten better. That has actually become more common; it has become less common for someone to come and say, ‘I’ve had dysuria since this morning.’ […]” (GP3, Pos. 2)*

Persistent symptoms despite previous self-treatment often shifted decisions toward antibiotic therapy, with the perceived adequacy of previous self-treatment also influencing individual accounts. The relative weight given to symptom burden, duration, and diagnostic findings varied between GPs. The threshold for antibiotic treatment was lower when symptoms raised concern for upper urinary tract involvement or systemic illness.

*“If people come in and say, ‘I can’t stand it anymore,’ and they have already been struggling with it for three weeks, and I also have, as discussed, a marked finding on the Combur test, then I treat it with antibiotics.” (GP15, Pos. 6)*

### Theme 2: Trust and established expectations could support or constrain antibiotic-sparing care

Familiarity and trust could facilitate antibiotic-sparing management. Several GPs described greater confidence in conservative treatment when they knew patients and trusted them to return if symptoms did not improve.

*“Of course, I ask whether they know this, whether they have had it before, and what helped them best. If someone comes for the first time and I don’t know her, I am more generous with antibiotics than if I already know her. If I already know her, I feel that the trust is there; she will come back if it does not get better.” (GP14, Pos. 40)*

GPs’ accounts suggested that previous treatment experiences could shape expectations. In their descriptions, successful non-antibiotic treatment appeared to facilitate subsequent use of such strategies, whereas patients perceived as accustomed to antibiotics or convinced that only antibiotics would help were described as more difficult to engage in an antibiotic-sparing approach. Concerns about pyelonephritis were likewise described as potentially affecting patients’ willingness to accept antibiotic-sparing management. GPs varied in how proactively they addressed this risk, but several described reassurance and safety-netting as ways of supporting patients in trying a non-antibiotic approach.

*“[…] What is still very important to me, and something I have also learned, is to always tell patients that the transition from a harmless bladder infection, ‘harmless’ in quotation marks, to a dangerous pyelonephritis is actually, fortunately, very, very rare, even if one forgoes the antibiotic. And that is a small sentence, but in my opinion, it can do a lot of good for patient acceptance when you take away their fear.” (GP10, Pos. 5)*

The possibility of longer symptom duration without immediate antibiotic treatment represented another trade-off in these discussions. GPs described patients as differing in their willingness to tolerate prolonged symptoms in order to avoid antibiotics, whereas others preferred antibiotic treatment in the expectation of faster symptom relief.

GPs often tried to negotiate these expectations through discussion and shared decision-making, but some described eventually prescribing antibiotics when patients remained strongly opposed to an antibiotic-sparing approach.

*“Then, broadly speaking, there are two types of patients. Some say, ‘I know this already; for me, only an antibiotic helps.’ Then shared decision-making becomes somewhat difficult. […] Very many patients are grateful for the approach of not having to take an antibiotic.” (GP10, Pos. 5)*

The feasibility of this negotiated approach also depended on communication. When GPs were uncertain whether patients understood what the antibiotic-sparing approach involved or when reassessment was needed, some described a lower threshold for prescribing antibiotics.

GPs also described patients’ expectations as being shaped beyond the individual consultation. Inconsistent messages or treatment approaches from other physicians could make antibiotic-sparing recommendations more difficult to communicate and sustain, and some GPs called for greater consistency among healthcare professionals as well as broader public awareness of non-antibiotic treatment options.

*“On the other hand, it becomes very difficult when other colleagues or specialists handle it differently. What’s needed is a consensus among physicians and a shift in societal thinking.” (GP9, Pos. 68)*

Views on tools to support decision-making and patient communication were mixed. Some GPs considered visual decision aids or easily accessible digital and multilingual patient information useful for supporting discussions, whereas others said they would be unlikely to use such tools or questioned whether written information would reach patients who were not already well informed.

### Theme 3: Practical constraints shaped both immediate and delayed prescribing decisions

Practical circumstances influenced management in different ways. Under substantial workload or time pressure, some GPs described being more likely to prescribe antibiotics, whereas others considered counselling about antibiotic-sparing strategies manageable within routine consultations. When access to reassessment was limited, such as before weekends, public holidays, or travel, several GPs adapted contingency planning by providing a backup prescription or antibiotic for use only if symptoms failed to improve, without necessarily changing the initial treatment decision. Costs were also raised as a constraint, particularly because some non-antibiotic products were not reimbursed.

*“In the emergency practice, it sometimes makes a difference, or when we have a high stress level. We are only human, and now and then it happens that we probably spend only two minutes explaining things, and then she says, ‘But I want that; that doesn’t work for me.’ […] It is much easier, much faster. We try not to do it, but if we have an anaphylaxis and three lacerations waiting, then we cannot spend 10, 15, or 20 minutes explaining things to them.” (GP5, Pos. 28)*

*“Yes, although, as I said, if they come just before the weekend or before a public holiday, I would perhaps simply give them the antibiotic or the prescription as a backup. Not that I would treat them because of that; I make the same decision. I would simply give it to them in case it doesn’t get better.” (GP2, Pos. 26)*

A more detailed overview of individual factors influencing treatment decisions, together with additional illustrative quotations, is provided in S5 Table.

### Theme 4: Practice staff shaped care through both formal routines and informal discretion

GPs’ accounts suggested that practice staff already shaped UTI care before and around the GP consultation, although the extent and nature of this involvement varied considerably between practices. Medical Practice Assistants were commonly described as a first point of contact and as contributing to triage and urine testing. In some practices, GPs also reported that they provided advice on fluid intake, analgesics, or other symptomatic measures, whereas in others their role was described as largely limited to preparing patients and diagnostic information for the physician. GPs also described variation in how these activities were organized: some reported agreed workflows or regular team-based teaching, while others described procedures that had evolved informally, without structured training or written protocols.

*“I can’t remember exactly [how it developed]. No, we did not really do any structured teaching. They know what they have to say, but, yes.” (GP3, Pos. 86)*

Even within more structured arrangements, staff experience and confidence seem to influence how telephone triage and counselling were handled. In some practices, particularly during periods of high workload, staff also initiated treatment-related decisions or sought rapid physician approval for treatment. A more detailed overview of current roles and activities is provided in S6 Table.

*“I give my MPAs [Medical Practice Assistant], and the MPK [Medical Practice Coordinator], who is the lead, a lot of freedom. When a patient comes in with symptoms for symptomatic treatment, they are allowed to advise her, for example on Femannose, Canephron [herbal remedy with rosemary leaves, centaury plant, and lovage root], or Angocin [herbal remedy with nasturtium and horseradish], we have all of that available, and they are also allowed to dispense it.”* (GP15, Pos. 44)

*“So, they don’t actually treat patients independently. It’s more like, sometimes when we don’t have any appointments, they’ll say, ‘Just come by anyway.’ They do the urine dipstick and then, kind of in passing, ask, ‘What should I dispense?’ (laughs). So, something like that. But treating patients on their own: no. They certainly give advice over the phone, though; for example, if someone calls, they might say, ‘Take some ibuprofen and drink plenty of fluids,’ and so on. But actually treating the patients themselves: no.”* (GP3, Pos. 80)

Advanced practice nurses were uncommon in the participating practices and were mainly involved in chronic care. One GP described Advanced practice nurses managing acute uncomplicated UTIs independently in selected cases, with physician approval when antibiotic treatment was required.

### Theme 5: Support for role expansion did not imply agreement on professional autonomy

Many GPs could envisage an expanded role for practice staff in managing uncomplicated UTIs and described potential benefits such as reducing physician workload and strengthening staff roles. Role expansion was not viewed solely as an efficiency gain. Some GPs valued seemingly simple consultations as opportunities to maintain patient contact and address preventive care, while patient acceptance, reimbursement, and available practice capacity were also seen as relevant conditions for implementation. However, views differed substantially regarding the degree of professional autonomy that should accompany such role expansion. Some considered independent management appropriate after sufficient training and clarification of the legal framework, whereas others preferred a delegated model with continued physician oversight, particularly when antibiotic treatment was considered. Others remained more sceptical, emphasizing variation in staff training and the clinical experience required to recognize patients who did not fit an uncomplicated UTI pathway. Perceived opportunities, concerns, and prerequisites for role expansion are summarized in S7 Table.

*“If I am responsible, then I would prefer them to consult me. Because somehow I have to take responsibility for the treatment. I give my staff a lot of freedom, but it does sometimes happen that things are assessed incorrectly.” (GP15, Pos. 60)*

*“The cases are so different, and of course there is a majority of uncomplicated cases, but there are also those that have to be filtered out, where a proper medical history shows that another problem is actually the main issue or that it is not uncomplicated after all. […] Training is one thing; the other is experience. They can read as much as they like or attend training courses, but the clinical experience is missing.” (GP9, Pos. 94-96)*

### Theme 6: Partial visibility shaped how GPs judged pharmacy-based care

GPs often had only partial visibility into pharmacy-based UTI care. Several reported limited knowledge of how pharmacy consultations were conducted, which criteria guided treatment decisions, or which antibiotics pharmacists could dispense. Limited visibility was not the only basis for GPs’ views of pharmacy-based care. Some questioned pharmacy-led UTI management because of perceived limitations in pharmacists’ access to complete medical histories and concerns about clinical training and experience. Others regarded pharmacists’ expertise as underused and welcomed a broader role in counselling, diagnosis, and treatment when appropriate structures were in place. Several GPs therefore called for clearer shared principles, referral criteria, and responsibilities between community pharmacies and general practices.

*“Well, they just don’t have the medical training for it, and above all, in most cases they also don’t have the patient’s full medical history and that’s really important. I know how it goes: patients say something, and then I ask, for example, ‘What about allergies?’ -‘No, no allergies.’ And then I say, ‘But it [the patient files] says here you have a penicillin allergy.’ ‘Ah, yes, that’s right.’ That’s how it is. They just can’t provide reliable information, and how are you supposed to assess that properly in a pharmacy?”* (GP12, Pos. 131)

*“[…] For me, this is fundamentally an area that I consider very important, and I am firmly convinced that a paradigm shift is needed in Switzerland. I find it unbearable, stupid, and short-sighted that we train pharmacists through an enormously demanding course of study only to have them hand out glucose samples afterwards. There is an incredible amount of expertise there that simply lies unused and is not being utilized. I would more than welcome pharmacists being given greater competencies in counselling, diagnostics, and therapy.” (GP10, Pos. 89)*

Contact was often indirect and mediated by patients, meaning that GPs were more likely to become aware of patients referred to them or those whose symptoms persisted, while successful pharmacy-based care could remain largely invisible.

*“That’s the thing. Even if they can dispense antibiotics and the patient goes to the pharmacy and then feels better afterwards, the problem is that I don’t hear about it at all. They have no reporting obligation of any kind to the GP. That’s the difficult part. I have no idea what’s going on. I don’t know which antibiotic they take, the treatment duration, or the dosage, because I simply don’t get that information. When the patient is healthy again, they just say, ‘Yes, the pharmacist helped me.’”* (GP13, Pos. 120)

Experiences nevertheless varied with local relationships: some GPs described little direct exchange, whereas others reported established relationships and direct communication with nearby pharmacists. Preferences for information exchange also differed; some wanted to be informed particularly when antibiotics were dispensed, whereas others considered routine case-by-case reporting unnecessary and instead wanted greater transparency about pharmacy decision pathways.

*“No, because with the two pharmacies we work with a lot, we have never talked about this at all. Otherwise, we do exchange information; we always call each other about prescriptions and things like that. But this topic has never come up between us.” (GP11, Pos. 138)*

## Discussion

In this exploratory study, six themes captured how antibiotic-sparing UTI management was shaped by the interaction of prescribing thresholds, relational expectations, and practical constraints, as well as by the roles and professional boundaries of other primary care providers. Across these themes, antibiotic-sparing care emerged as a negotiated and context-dependent process rather than a simple treatment choice. The findings also indicate that decisions are influenced not only within the GP consultation itself, but across the broader care pathway, including through the involvement of practice staff and community pharmacies. Taken together, antibiotic-sparing UTI management therefore extends beyond the individual GP prescribing decision and is shaped by how responsibilities, communication, and expectations are distributed across primary care.

### Determinants and challenges of implementing antibiotic-sparing strategies in general practice

Antibiotic-sparing strategies were widely recognized among participants, with many GPs reporting that they already manage uncomplicated UTI cases without antibiotics. Many of the factors identified in this study correspond to those previously reported in the literature as potentially influencing therapeutic decision-making in uncomplicated UTIs [7], encompassing a broad spectrum of clinical, contextual, and patient-related dimensions.

Our findings are consistent with a recent qualitative evidence synthesis showing that UTI management in primary care is shaped not only by clinical considerations, but also by healthcare-system constraints and the clinician–patient relationship [8]. While some factors are not modifiable, others, including patient expectations or organizational conditions, offer opportunities for targeted intervention. Patient demand for antibiotics remained a prominent challenge [18], particularly in situations of high workload or before weekends and holidays. Addressing these modifiable factors may enable GPs to apply antibiotic-sparing approaches more consistently and confidently, whenever clinically appropriate.

Another finding of this study concerns how GPs address the potential limitations of antibiotic-sparing strategies in everyday practice. The extent to which the risks most frequently cited in the literature, such as a slightly increased likelihood of pyelonephritis or prolonged symptom duration [1,7], was discussed varied between GPs. Some addressed it proactively, whereas others focused primarily on warning signs and safety-netting or discussed the risk in more detail when patients expressed concerns. This variation may reflect the role of pragmatic, experience-based reasoning in everyday primary care. Such reasoning has been described as relying on “mindlines”, that is, collectively reinforced and tacit knowledge shaped by clinical experience and routine practice, rather than exclusively on formal guideline recommendations [19]. GPs who perceived progression to pyelonephritis as uncommon in their own practice may therefore have regarded detailed risk discussions as less relevant, while previous experience with complications could make the issue more salient. At the same time, some GPs described reassurance about the low absolute risk of progression as a way of reducing patients’ concerns and supporting acceptance of an antibiotic-sparing strategy.

In addition, our findings suggest previous unsuccessful self-treatment may leave less scope for a further trial of symptomatic management at the GP consultation. Current guidelines discourage symptomatic management once symptoms have persisted for more than five days [20]. As several GPs reported, delayed consultation is not uncommon and often follows unsuccessful self-care attempts [21,22]. This delay may narrow the therapeutic window for proactive non-antibiotic management and reduce the number of patients eligible for an antibiotic-sparing strategy.

### Potential and preconditions for greater involvement of non-physician medical staff

Regarding the role of non-physician medical staff, our findings suggest that their contribution to the management of uncomplicated UTIs already extends beyond purely administrative or preparatory tasks, although these roles are often embedded in locally developed and partly informal routines. Regardless of their current scope of practice, non-physician medical staff occupy an important position within general practice, and emerging evidence suggests that they can contribute to antimicrobial stewardship through patient communication, care navigation, and the implementation of practice-wide stewardship activities [23,24]. Their involvement therefore has several dimensions: From a clinical perspective, greater participation in the management of uncomplicated UTIs could improve efficiency, alleviate physicians’ workload, and enhance patient access to timely care. Professionally, an expanded scope may increase the attractiveness and long-term retention within the profession by providing opportunities for skill development and recognition. From an antimicrobial stewardship perspective, practice staff are well positioned to promote consistent principles through their frequent and trusted contact with patients, ensuring coherent communication and reducing the risk of unintentionally priming patients toward expecting antibiotics. Whether a more active involvement of practice staff in patient management is meaningful appears to depend on individual and contextual factors such as professional role perception, local workload, or physician shortages. Importantly, openness toward role expansion did not necessarily imply agreement on the appropriate degree of professional autonomy, highlighting a tension between the perceived practicality of delegation and physicians’ desire to maintain oversight and ensure quality of care. GPs also emphasized the need for robust training, legal clarity, reimbursement, and patient acceptance, barriers that have also been described in previous studies on antimicrobial stewardship and nursing [9,10,25–27]. While the exploratory nature of this study does not allow for definitive conclusions, the findings indicate potential for a stronger integration of practice staff in the management of uncomplicated UTIs, provided that appropriate frameworks, training, and support are in place. This is particularly relevant in Switzerland, where task shifting from GPs to practice assistants and nurses was less pronounced than in many other European countries in an international survey [28], suggesting opportunities to better utilize the skills and capacities of practice staff.

### GP–Pharmacy collaboration and professional interfaces

Dispensing antibiotics for uncomplicated UTIs without a physician’s prescription has been introduced in several regions of the UK, the USA, Canada, and Australia [29]. In the 2024 Swiss population survey, 3% of respondents who had taken antibiotics in the preceding 12 months reported obtaining their most recent course from a pharmacy without a physician’s prescription [30], and decision trees are available for the management of various minor conditions, including uncomplicated UTI [12].

Against this background, our findings suggest that GPs’ perceptions of pharmacy-based care need to be interpreted in light of their often limited and selective visibility into these care pathways. When successfully managed cases remain largely outside general practice, while patients with persistent symptoms or complications subsequently present to GPs, professional judgments may be disproportionately informed by cases of non-resolution. This asymmetry in visibility may contribute to uncertainty or reservations toward pharmacy-led care, even in the presence of growing evidence supporting pharmacist-led management in other healthcare settings [11].

At the same time, the findings suggest that perceptions of pharmacy-based care are shaped not only by information exchange, but also by local professional relationships and broader views of professional roles and boundaries. Greater transparency regarding pharmacy decision pathways, referral criteria, and responsibilities may therefore be important not only for continuity of care, but also for establishing mutual trust and a shared understanding of respective professional roles [31]. This may be particularly relevant given that views on an expanded role for pharmacists ranged from concerns about clinical responsibility and access to medical information to recognition of pharmacists’ expertise as an underused resource within primary care. An additional structural factor is that a number of patients do not have a regular general practitioner. In such cases, community pharmacies may function as a primary point of contact, and expectations of routine bidirectional communication with general practice may not be readily applicable. Rather than requiring communication for every pharmacy encounter, feasible mechanisms for information exchange may therefore need to focus particularly on situations in which continuity of care is relevant, such as referral to general practice or the dispensing of prescription-only medication.

### Implications for future research

Future research should aim to better understand the modifiable factors that shape GPs’ treatment decisions. Gaining insight into these factors will require directly incorporating the patient perspective, including exploring how women interpret symptoms, navigate self-management options, and decide when to seek care. Building on this, research should examine patient trajectories from symptom onset to pharmacy encounters and eventual presentation in general practice, to identify the points at which antibiotic-sparing strategies are feasible and how acceptance of non-antibiotic treatment can be supported. Further work is also needed to determine which competencies, decision-support structures, and training elements enable non-physician medical staff to safely and effectively contribute to uncomplicated UTI management. Finally, enhancing continuity of care across health care professionals will require research on feasible and low-burden communication mechanisms between GP practices and community pharmacies, as improved information exchange and mutual understanding of decision-making processes could support safer and more coordinated management of uncomplicated UTIs thus enhancing antimicrobial stewardship by strengthening interprofessional primary care.

### Strengths and limitations

A major strength of this study lies in its exploratory design, which allowed for an in-depth examination of a complex and understudied topic. The inclusion of a heterogeneous group of GPs with varying levels of experience, regional backgrounds, and practice models (including self-dispensing practices) enabled a broad range of perspectives. The study focused on a highly relevant clinical issue that all participants regularly encounter in daily practice, ensuring the practical applicability of our findings. The use of a semi-structured interview guide provided both structure and flexibility, facilitating comparability across interviews while allowing new themes to emerge. Finally, the study contributes novel insights into antimicrobial stewardship and interprofessional collaboration within the unique context of the Swiss healthcare system.

A potential limitation of this study is the relatively small sample size of 15 GPs. However, no substantively new content relevant to the research questions emerged during the later interviews. This may be explained by the focused and practice-oriented nature of the research questions and the structured interview guide, which directed participants toward key aspects of uncomplicated UTI management. The topic itself was highly relevant and familiar to all participants, as uncomplicated UTIs represent a common condition in daily general practice. Another limitation is that all participating GPs were accredited teaching physicians affiliated with the university. This may have introduced selection bias, as teaching physicians may differ from the broader GP population in ways that were not captured in this study, thereby limiting the transferability of the findings. Additionally, the study focused on the GP perspective; consequently, the views of community pharmacists, non-physician medical staff, and patients were not captured. Interpretations of GP–pharmacy collaboration therefore reflect professionally situated perceptions rather than an objective assessment of interprofessional practice. While the findings may not be representative for all GPs, they provide valuable insights into current practice and may inform future research and implementation efforts. Future studies in broader and more diverse populations could examine the transferability of these findings and quantify selected patterns where appropriate.

## Conclusion

This exploratory study highlights the complexity of managing uncomplicated UTIs in general practice and underscores the interplay of clinical, relational, contextual, and interprofessional factors shaping antibiotic-sparing decisions. While GPs generally aimed to use antibiotic-sparing strategies, their implementation remained conditional and was influenced by patient expectations, trust, and practical constraints. Practice staff already contributed to UTI management, although GPs differed in their views on the appropriate degree of professional autonomy. Pharmacy-based care was often only partly visible to GPs, with experiences varying according to local relationships and information exchange. Clearer roles, appropriate training, and feasible communication structures may support more coordinated antibiotic-sparing care and antimicrobial stewardship in primary care. Overall, the findings indicate that antibiotic-sparing UTI management is shaped not only at the point of prescribing but across the broader primary care pathway.

**Use of generative artificial intelligence:** Generative artificial intelligence (ChatGPT 5.6, OpenAI) was used to support English-language editing, as the authors are not native English speakers, and to check the internal consistency of the manuscript, including terminology, wording, cross-references, and presentation across sections. The tool was not used to generate or collect study data. All AI-assisted suggestions were critically reviewed by the authors, and all analytical decisions, interpretation of the findings, and final wording remained the responsibility of the authors. The authors take full responsibility for the content of the manuscript.

## Supporting information

S1 Table Predefined thematic framework guiding the semi-structured interviews. (DOCX)

S2 Table Interview guide for general practitioners. (DOCX)

S3 Table Code list and frequency of code segments. (DOCX)

S4 File COREQ checklist. (DOCX)

S5 Table Factors influencing GPs’ treatment decisions in uncomplicated UTIs. (DOCX)

S6 Table Roles and activities of Medical Practice Assistants and Medical Practice Coordinators in the management of patients with suspected urinary tract infection. (DOCX)

S7 Table Perceived opportunities, concerns, and prerequisites for expanding the roles of Medical Practice Assistants and Medical Practice Coordinators in uncomplicated UTI management. (DOCX)
