# Supplementary material for: General practitioners’ perspectives on treatment decisions and interprofessional care for women with uncomplicated urinary tract infections: An exploratory qualitative study
Source: PLoS One. 2026 Nov 11;21(11):e0362328. doi: 10.1371/journal.pone.0362328 (PMC13754150; doi:10.1371/journal.pone.0362328)
Supplement: Supplementary file 1 [file pone.0362328.s001.docx]

**Table S1:** Predefined thematic framework guiding the semi-structured interviews

| Interview part | Domain |
| --- | --- |
| Decision-making in patients with uncomplicated UTI | - Experience with antibiotic-sparing strategies - Decision-making criteria - Barriers to using antibiotic-sparing strategies - Facilitators / enabling factors |
| The role of the non-physician practice staff | - Current roles of MPAs/MPKs/APNs - Acceptance of task delegation / role expansion - Barriers to role expansion - Opportunities / potential of role expansion |
| Interprofessional collaboration with community pharmacies | - Level of information about pharmacy services - Experience with pharmacy collaboration - Needs & expectations for interprofessional collaboration - Preferences for information exchange / communication |
| APN: Advanced practice nurse; MPA: medical practice assistants; MPK: Medical Practice Coordinators; UTI: Uncomplicated Urinary Tract Infection | |
