# Supplementary material for: General practitioners’ perspectives on treatment decisions and interprofessional care for women with uncomplicated urinary tract infections: An exploratory qualitative study
Source: PLoS One. 2026 Nov 11;21(11):e0362328. doi: 10.1371/journal.pone.0362328 (PMC13754150; doi:10.1371/journal.pone.0362328)
Supplement: Supplementary file 2 [file pone.0362328.s002.docx]

**Supplemental table S2: Interview Guide for General Practitioners**

**Section 1: Treatment decisions in uncomplicated urinary tract infections**

**Introduction / Background:** Uncomplicated urinary tract infections (uUTIs) are usually self-limiting. The standard of care has traditionally been antibiotic therapy, primarily aimed at symptom control. However, given rising antimicrobial resistance rates, antibiotic-sparing approaches have gained increasing attention.

Uncomplicated UTIs are defined as acute bladder infections (cystitis) in otherwise healthy, non-pregnant women aged 18–64 years, often specified as occurring “without functional or anatomical abnormalities of the urinary tract.”

The national guidelines of the Swiss Society for Infectious Diseases recommend antibiotic-sparing treatment (ASPT) for patients with uUTIs. The core idea is to treat patients symptomatically first. Optionally, a prescription for antibiotics can be provided for self-initiation in case of non-improvement, or the patient can return for reassessment and receive antibiotics if symptoms persist.

**Main question:** Have you already gained experience with ASPT? What does this experience look like?

**Follow-up questions (optional)**

- Which forms of ASPT do you apply, and why? (e.g., Ibuprofen, D-mannose, Cranberries; delayed prescription, follow-up visits)

- Where did you learn about these options? Guidelines? Literature? Peers? Quality circles?

- Do patients treated with ASPT return to your practice?

- Have you ever seen a case of pyelonephritis following ASPT?

**Main question:** How do you decide whether to treat a patient with antibiotics immediately or to use an antibiotic-sparing approach?

**Follow-up questions (optional)**

- Are there any patient groups for whom you would generally not consider ASPT?

- How do the following factors influence your decision?

- Urine dipstick results (e.g., negative but typical symptoms, only leukocytes or nitrite positive)

- Duration and severity of symptoms

- Familiarity with the patient

- Patient preferences and previous treatment decisions

- Timing of consultation (e.g., before weekends or holidays)

- Patient age, educational or migration background

- Structural or economic aspects

**Main question:** Are there factors or circumstances that prevent or make it difficult for you to use ASPT more often?

**Follow-up questions (optional)**

- Do patients tend to present late to the GP?

- Have patients often already started self-treatment?

- Do patients explicitly request antibiotics?

- Are there knowledge or language barriers?

- Are there structural constraints, such as time pressure?

- Is antimicrobial resistance (AMR) perceived as irrelevant?

- Concerns about risk of pyelonephritis or longer symptom duration?

**Main question:** From your perspective, what would help you to use ASPT more frequently in patients with uncomplicated UTIs?

**Follow-up questions (optional)**

- Information materials for patients (e.g., flyers, posters, videos)?

- Decision-making aids for consultations?

- Better-known or more accessible GP guidelines?

**Section 2: Interprofessional care within general practice**

**Introduction / Background**: The competencies of medical practice assistants/coordinators (MPAs/MPKs) and advanced practice nurses (APNs) in ambulatory care are expanding, particularly in chronic disease management and prevention. Their involvement can improve care quality, resource allocation, and the attractiveness of primary care professions.

Uncomplicated UTIs are common, simple to treat, and generally associated with a favourable prognosis. This raises the question of whether MPAs, MPKs, or APNs could in the future manage such cases, either under delegation or autonomously.

**Main question**: What role do MPAs, MPKs, or APNs currently play in your practice in managing patients with suspected UTIs?

**Follow-up questions (optional)**

- What exactly do MPAs/MPKs/APNs do in this context?

- Do they perform urine dipstick tests for relevant patients?

- Do they provide telephone counselling or triage?

- Are any tasks already delegated or partially independent?

**Main question**: Assuming appropriate legal frameworks exist: Could you imagine MPKs or APNs managing patients with suspected uncomplicated UTIs, either under delegation or autonomously?

**Follow-up questions (optional)**

- Would you welcome an expansion of these competencies? Why or why not?

- Would you consider implementing this in your own practice?

**Main question**: Where do you see barriers to expanding these competencies?

**Main question**: Where do you see opportunities associated with expanding these competencies?

**Section 3: Interprofessional collaboration between GPs and pharmacies**

**Introduction / Background**: An increasing number of community pharmacies now offer consultations and treatment for patients with urinary symptoms. Pharmacists may provide symptomatic therapy and, under specific conditions, dispense prescription-only medications, including antibiotics, without a physician’s prescription.

**Additional information (if needed)**: This requires a “justified exceptional case.” In addition, pharmacies may dispense predefined medications listed on “List B Plus,” which does not include antibiotics. For UTIs, antibiotics are therefore only dispensed under this justified exception.

**Main question**: How well informed do you feel about pharmacy-based services for uUTI management?

**Follow-up questions (optional)**

- Do you know how such consultations are conducted?

- Do you know what criteria are used to decide on treatment or antibiotic use?

- Do you know which antibiotics pharmacists are allowed to dispense?

**Main question**: Have you had any experience or contact with pharmacies treating patients with UTIs?

**Follow-up questions (optional)**

- Who initiated the contact?

- How did the communication take place (e.g., phone, email, fax)?

- What worked well, and what did not?

- Have you had patients referred from pharmacies or reporting previous consultations there?

- Do you routinely ask whether patients visited a pharmacy first?

**Main question:** What would you consider important for good interprofessional collaboration with pharmacies in managing patients with acute uncomplicated UTIs?

**Main question (if not already addressed)**: Would you, as a GP, welcome being informed by pharmacies about the counselling or treatment of your patients?

**Follow-up questions (optional)**

- In which cases would you want to be informed?

- What kind of information would you expect?

- Through which communication channels (e.g., phone, email, fax, SMS)?
