# Supplementary material for: General practitioners’ perspectives on treatment decisions and interprofessional care for women with uncomplicated urinary tract infections: An exploratory qualitative study
Source: PLoS One. 2026 Nov 11;21(11):e0362328. doi: 10.1371/journal.pone.0362328 (PMC13754150; doi:10.1371/journal.pone.0362328)
Supplement: Supplementary file 3 [file pone.0362328.s003.docx]

**Supplemental table S3: Code list and frequency of code segments**

| **List of Codes** | **Frequency** |
| --- | --- |
| Overall codes segments | 1547 |
| Coding domain 1 | 0 |
| Patient characteristics | 3 |
| Patients demand | 8 |
| Late first consultation | 1 |
| previous therapies | 14 |
| Outlook for more AST | 0 |
| Guidelines | 11 |
| Decision-making aids | 7 |
| Information for patients | 39 |
| Decision-making | 0 |
| Influence of antibiotic resistance on decision-making | 18 |
| Influence of shared decision-making | 8 |
| Influence of Pyelonephritis on decision-making | 30 |
| Influence of patients’ self-treatment on decision-making | 29 |
| Influence of the frequency of urinary tract infections on decision-making | 11 |
| Influence of economic and structural aspects on decision-making | 49 |
| Influence of the patient's educational/migration background on decision-making | 22 |
| Influence of the patient's age on decision-making | 23 |
| Influence of the timing of consultation on decision-making | 31 |
| Influence of previous treatment decisions in earlier episodes of urinary tract infection on decision-making | 17 |
| Influence of the patient's wishes on decision-making | 46 |
| Influence of ‘knowing’ the patient on decision-making | 21 |
| Influence of the severity of local symptoms on decision-making | 35 |
| Influence of symptom duration on decision-making | 46 |
| Influence of diagnostic on decision-making | 57 |
| Experiences towards AST | 18 |
| Knowledge gaps (doctor) | 3 |
| Safety netting | 21 |
| Prevention of urinary tract infections | 10 |
| Frequency of urinary tract infection | 3 |
| Complications | 22 |
| Source of knowledge | 17 |
| Therapy failure | 25 |
| Own information gain | 6 |
| Types of AST | 80 |
| Coding domain 2 | 0 |
| Outlook for delegated or independent treatment by MPAs, MPKs or APNs | 0 |
| Enhancing the professional image | 7 |
| simple work, best point to start | 6 |
| Requirements for expanding competencies | 60 |
| Loss of simple cases | 4 |
| Delegated or independent? | 14 |
| Loss of patient contact | 3 |
| Opportunities for expanding competencies | 33 |
| Barriers to expanding competencies | 19 |
| Welcoming the expansion of competencies | 46 |
| Role of MPAs, MPKs or APNs | 4 |
| Development of the process | 17 |
| Fixed sequence of tasks | 19 |
| Independent/delegated work | 31 |
| Exact duties of MPAs, MPKs or APNs | 5 |
| Anamnesis | 13 |
| Medicaments | 15 |
| Labor | 7 |
| Advice | 39 |
| Urine culture | 2 |
| Decisions | 9 |
| Vital signs | 2 |
| Triage | 20 |
| Urine dipstick | 29 |
| Coding domain 3 | 0 |
| Welcoming counselling and therapy in pharmacies | 25 |
| Expectation of good interprofessional cooperation | 0 |
| Clear boundaries of pharmacies | 12 |
| Lack of communication | 33 |
| Desired information channel | 12 |
| Content of the information exchange | 15 |
| Trigger for information exchange | 21 |
| Experience with treatment and advice on urinary tract infections in pharmacies | 23 |
| Points of contact with pharmacies | 24 |
| Successful treatments by the pharmacy | 10 |
| Reporting by pharmacies | 5 |
| Profit of pharmacies | 14 |
| Number of pharmacy referrals | 13 |
| Pharmacy referral | 47 |
| Evaluation of contacts | 12 |
| Information channel for contacts | 2 |
| Contact initiation | 9 |
| Knowledge about the context in pharmacies | 3 |
| General practitioners' lack of knowledge about pharmacies | 21 |
| Antibiotics pharmacies | 18 |
| Criteria for treatment decisions | 14 |
| Consultation process | 16 |
