# Supplementary material for: General practitioners’ perspectives on treatment decisions and interprofessional care for women with uncomplicated urinary tract infections: An exploratory qualitative study
Source: PLoS One. 2026 Nov 11;21(11):e0362328. doi: 10.1371/journal.pone.0362328 (PMC13754150; doi:10.1371/journal.pone.0362328)
Supplement: Supplementary file 4 [file pone.0362328.s004.docx]

**Supplemental Table S4: COREQ checklist**

Developed from:

Tong A, Sainsbury P, Craig J. Consolidated criteria for reporting qualitative research (COREQ): a 32-item checklist for interviews and focus groups. International Journal for Quality in Health Care. 2007. Volume 19, Number 6: pp. 349 – 357

| **Item No** | **Guide Questions/Description** | **Reported on Page #** |  |  |
| --- | --- | --- | --- | --- |
| **Domain 1: Research team and reflexivity** | | |  |  |
| **Personal Characteristics** | | |  |  |
| 1. Interviewer/ facilitator | Which author/s conducted the interview or focus group? | Pg 8 |  |  |
| 2. Credentials | What were the researcher’s credentials? E.g., PhD, MD | Title page |  |  |
| 3. Occupation | What was their occupation at the time of the study? | Title page |  |  |
| 4. Gender | Was the researcher male or female? | male |  |  |
| 5. Experience and training | What experience or training did the researcher have? | AP is a physician specialized in general internal medicine and infectious diseases and an experienced primary care researcher. TT was a master’s student who received training and guidance in qualitative research, transcription, and coding. See Methods. |  |  |
| **Relationship with participants** | | |  |  |
| 6. Relationship established | Was a relationship established prior to study commencement? | Some participants were known to the interviewer (AP) through previous professional contact at the Institute of Primary Care. This familiarity may have contributed to a more open discussion atmosphere. |  |  |
| 7. Participant knowledge of the interviewer | What did the participants know about the researcher? e.g. personal goals, reasons for doing the research? | Participants were informed about the professional background of the interviewer (AP), who is a Specialist in General Internal Medicine and Infectious Diseases and researcher at the Institute of Primary Care. The interviewer’s research focuses on infections, antibiotic use, and antibiotic consumption in primary care. Few participants were personally acquainted with the interviewer and were therefore already aware of this professional focus. |  |  |
| 8. Interviewer characteristics | What characteristics were reported about the interviewer/facilitator? e.g. Bias, assumptions, reasons and interests in the research topic | AP has a clinical background in general internal medicine and infectious diseases and a research focus on infections and antibiotic use in primary care. This background may have influenced interview probing and interpretation of the data. Reflexivity was addressed through discussion within the analytic team and consideration of divergent accounts. |  |  |
| **Domain 2: study design** | | |  |  |
| **Theoretical framework** | | |  |  |
| 9. Methodological orientation and Theory | What methodological orientation was stated to underpin the study? e.g. grounded theory, discourse analysis, ethnography, phenomenology, content analysis | Thematic analysis informed by Braun and Clarke’s framework was used. The analysis combined deductive and inductive elements: predefined interview domains provided an initial analytical structure, while additional codes were developed inductively from the data. |  |  |
| **Participant selection** | | |  |  |
| 10. Sampling | How were participants selected? e.g., purposive, convenience, consecutive, snowball | Pg 7 |  |  |
| 11. Method of approach | How were participants approached? e.g., face-to-face, telephone, mail, email | Pg 7 |  |  |
| 12. Sample size | How many participants were in the study? | Pg 11 |  |  |
| 13. Non-participation Setting | How many people refused to participate or dropped out? Reasons? | The number of invited GPs who declined or did not respond was not systematically recorded. |  |  |
| 14. Setting of data collection | Where was the data collected? e.g., home, clinic, workplace | Pg 7 |  |  |
| 15. Presence of nonparticipants | Was anyone else present besides the participants and researchers? | No |  |  |
| 16. Description of sample | What are the important characteristics of the sample? e.g. demographic data, date | Table 1 |  |  |
| **Data collection** | | |  |  |
| 17. Interview guide | Were questions, prompts, and guides provided by the authors? Was it pilot tested? | Supplemental Table S2, Pg 7 |  |  |
| 18. Repeat interviews | Were repeat interviews carried out? If yes, how many? | no |  |  |
| 19. Audio/visual recording | Did the research use audio or visual recording to collect the data? | Pg 7 |  |  |
| 20. Field notes | Were field notes made during and/or after the interview or focus group? | Yes – during the first interviews, to improve the interview guide |  |  |
| 21. Duration | What was the duration of the interviews or focus group? | Table 1 |  |  |
| 22. Data saturation | Was data saturation discussed? | The recruitment stopping criterion is described in the Methods and Results: additional interviews were planned until no substantively new content relevant to the research questions emerged. |  |  |
| 23. Transcripts returned | Were transcripts returned to participants for comment and/or correction? | no |  |  |
| **Domain 3: analysis and findings** | | |  |  |
| **Data analysis** | | |  |  |
| 24. Number of data coders | How many data coders coded the data? | Three researchers (AP, NH, and TT) contributed to the initial coding and development of the coding framework. TT served as the primary coder. Subsequent coding and analysis were divided between TT and AP, with TT focusing primarily on the second interview domain. AP reviewed and revised the coding across the complete dataset. NH contributed primarily to the initial development and refinement of the coding framework.  The coding framework was reviewed and refined again after approximately half of the remaining transcripts had been coded before being applied to the complete dataset. |  |  |
| 25. Description of the coding tree | Did the authors provide a description of the coding tree? | Yes, supplemental file |  |  |
| 26. Derivation of themes | Were themes identified in advance or derived from the data? | Initial coding combined predefined interview domains with inductively generated codes. After the initial coding and descriptive organization of the findings, the coded dataset was subsequently revisited to identify cross-cutting patterns of shared meaning. These patterns were developed into candidate themes and iteratively refined against the coded data. |  |  |
| 27. Software | What software, if applicable, was used to manage the data? | Pg 8 |  |  |
| 28. Participant checking | Did participants provide feedback on the findings? | No |  |  |
| **Reporting** | | |  |  |
| 29. Quotations presented | Were participant quotations presented to illustrate the themes/findings? Was each quotation identified? e.g., participant number | yes |  |  |
| 30. Data and findings consistent | Was there consistency between the data presented and the findings? | yes |  |  |
| 31. Clarity of major themes | Were major themes clearly presented in the findings? | Six major themes are clearly presented and described in the Results section. |  |  |
| 32. Clarity of minor themes | Is there a description of diverse cases or a discussion of minor themes? | Variation between participants, including divergent and contradictory accounts, is described within the individual themes. |  |  |
