# Supplementary material for: General practitioners’ perspectives on treatment decisions and interprofessional care for women with uncomplicated urinary tract infections: An exploratory qualitative study
Source: PLoS One. 2026 Nov 11;21(11):e0362328. doi: 10.1371/journal.pone.0362328 (PMC13754150; doi:10.1371/journal.pone.0362328)
Supplement: Supplementary file 5 [file pone.0362328.s005.docx]

| **Supplemental Table S6: Factors influencing GPs’ treatment decisions in uncomplicated UTIs** | |
| --- | --- |
| **Factors** | **Quotes** |
| **Symptom burden:** Mild presentations were more likely to be managed with antibiotic-sparing strategies, whereas severe symptoms or signs of systemic involvement led to antibiotic prescribing.  Contextual factors, such as occupational demands, could further affect decisions. | - *“I say that if the pain is not severe and the urine is not visibly bloody, then I recommend a trial of treatment with NSAIDs.”* (GP14, Pos. 4) - *“Then I really base it on the symptoms, yes. So, if people come in and say, ‘I can’t stand it anymore,’ …. then I treat it with antibiotics.”* (GP15, Pos. 6) - *“And the level of distress at work, for example, a saleswoman or someone in the service sector who says, ‘I can’t go to the toilet every five minutes,’ or something like that.”* (GP11, Pos. 26) |
| **Duration of symptoms:** Shorter symptom duration favoured antibiotic-sparing treatment, whereas prolonged symptoms increased the likelihood of antibiotic therapy, particularly when accompanied by higher symptom burden. | - *“Yes, I do make a distinction. If someone comes in and convincingly tells me, ‘I’ve had these symptoms for five, six, seven days now, they’re not getting any better, or even worse. I’ve really been drinking a lot, I’ve taken painkillers, maybe cranberry juice, and still nothing has helped,’ then, if I have a corresponding urine finding, at some point I would start treatment.”* (GP1, Pos. 36) - *“Yes, if she has already treated herself for five days and it hasn’t gotten any better, then I also prescribe antibiotics.”* (GP3, Pos. 20) |
| **Patient demand:** Patient demand for antibiotics was frequently reported. Patient expectations strongly shaped GPs’ decisions, especially when patients were convinced they needed antibiotics. Some GPs complied with such requests to preserve the doctor–patient relationship or to prevent patients from seeking antibiotics elsewhere. High workload or time pressure further increased the likelihood of yielding to patient demand. Others, however, actively engaged in education and offered alternatives, especially when they knew the patient well. | - *“… there are two types of patients: some say, ‘I know this already, only an antibiotic helps me.’ In those cases, shared decision-making becomes rather difficult, …”* (GP10, Pos. 5) - *“No, I also point out that in the past we used to give antibiotics more readily, and that nowadays we try a different approach, asking whether they would agree to give that a try.”* (GP14, Pos. 48) - *“Exactly, skeptical toward the antibiotic-sparing approach. It’s the classic case: the woman who says, ‘I know this already; it never works without antibiotics for me.’ And if I manage to convince her to try ibuprofen instead, those are disproportionately often the patients who come back after two or three days saying, ‘Now I do need the antibiotic - it’s still not better.’”* (GP10, Pos. 9) - *“I find it relatively difficult because patients are very focused on antibiotics. In my experience, there is a group of patients where you can say whatever you want, in the end, they want the antibiotic and insist on getting it.”* (GP1, Pos. 2) |
| **Previous self-management:** Previous unsuccessful self-treatment could shift decisions toward antibiotics; in some accounts, the perceived adequacy of that treatment also mattered. | - *“I have many patients who come only after two or three days, having already treated themselves symptomatically without improvement. If we then have a positive urine dipstick, we would, of course, treat them with antibiotics.”* (GP9, Pos. 32) - *“If they’ve really done that well and have actually taken their NSAIDs three times a day and the D-mannose in a high dose, well then, I’m more likely to give them an antibiotic.”* (GP4, Pos. 58) |
| **Timing of consultation:** Consultations immediately before weekends, holidays, or patient travel often led to the provision of antibiotics or backup prescriptions, motivated by concerns about access to care and symptom control during these periods. Time pressure, especially on Friday afternoons or in emergency settings, further influenced this practice. | - *“Yes, if someone is going on vacation or comes in over the weekend, then I sometimes give them the antibiotic and say that if it doesn’t respond to the treatment attempt with an anti-inflammatory medication, then she may take the antibiotic, and afterward she should call to ask about the result of the urine culture.” (GP14, Pos. 60)* |
| **Previous treatment decisions**: Past treatment experiences influenced both physicians and patients. Positive outcomes with antibiotic-sparing treatment encouraged GPs to repeat such strategies, while some patients referred to earlier prescriptions to question a current recommendation against antibiotics. | - *“Of course, it’s particularly nice to have patients who have already had good experiences with this. They know that it can work.”* (GP14, Pos. 72) - *[On previous treatment decisions with an antibiotic prescription] “That backfires sometimes. When [I] was too generous once, or didn’t have time, or I don’t even know. That always backfires.”* (GP6, Pos. 36) - *“We even take the time to educate patients who are hard to convince and say: ‘that never helps me,’ even though the tariff [of the consultation] doesn’t cover it.”* (GP5, Pos. 22) |
| **Doctor–patient relationship:** Familiarity with the patient influenced decision-making. Long-standing patients often knew their GP’s approach, which could facilitate antibiotic-sparing treatment. Conversely, GPs sometimes prescribed antibiotics more readily to maintain the therapeutic relationship. | - *“In the past five years, I’ve somewhat trained my patients so that they know they should take ibuprofen and drink plenty of fluids. Now, some of them even come in having already taken ibuprofen and increased their fluid intake before seeing me.”* (GP8, Pos. 8) |
| **Patients from abroad / migration status / health literacy:** Some GPs observed differing expectations among patients with migration backgrounds or from abroad, often shaped by experiences in their home countries. These consultations were reported to require more time, and communication difficulties could lead to a tendency toward antibiotic prescribing. | - *“If communication is more difficult and I get the feeling that someone may have more trouble understanding, I’m more likely to give the antibiotic.”* (GP14, Pos. 54) - *“Well, we have patients from all countries, so it’s the same for everyone. I do know that in certain southern countries people tend to complain a bit more, but in the end, it depends on the actual findings, and then you just have to figure out how to explain it to them.”* (GP13, Pos. 42) - *“So it depends on how well someone speaks German and understands what I’m saying. If I get the feeling that the person doesn’t understand anything at all or speaks very poor German and doesn’t grasp that she has to come back if she develops certain symptoms, then it’s obviously extremely difficult. In such cases, I would probably tend to prescribe antibiotics.”* (GP12, Pos. 35) |
| **Economic and structural aspects**: Although structural or financial considerations rarely played a major role, time constraints were occasionally mentioned. Limited consultation time sometimes prevented detailed discussions of antibiotic-sparing strategies, thereby favoring antibiotic prescription. GPs differed in their perceptions of the time required for history taking and counselling in uncomplicated uUTIs. Some considered it minor, while others described it as substantial, particularly when explaining non-antibiotic approaches or addressing patient expectations. In addition, several GPs noted that alternative or supportive products, such as phytotherapeutic remedies, are not reimbursed by health insurance, which may discourage their recommendation and reduce patients’ willingness to use them. | - *“Yes, there are quite a few of them [alternative products] - the problem is really that most of these products are not covered by health insurance and are relatively expensive.”* (GP15, Pos. 2) - *“I also need a bit of time for this explanation. When I’m under a lot of time pressure, I tend to give an antibiotic a bit more quickly. Yes, because if I don’t give one, I feel like I have to explain things in more detail. So, the time factor can occasionally play a role, yes.” (GP14, Pos. 58)* - *“Of course, when the consultation schedule is completely full, you’re naturally more inclined at some point to give in and not discuss it in such detail.”* (GP1, Pos. 34) |
| **Patient age:** For most GPs, patient age alone did not affect treatment choice. However, some were more inclined to prescribe antibiotics to older patients, citing comorbidities, polypharmacy (e.g., use of NSAIDs), or a perceived higher risk of complications. | - *“Many of them are already taking some kind of NSAID, at least as needed - they all have some osteoarthritis somewhere. Then we have to see: if someone is already taking something three times a day that they bought themselves, then I’m more inclined to decide to give an antibiotic. I can’t just say, let’s increase from 400 mg to 800 mg of ibuprofen. That doesn’t make sense.”* (GP13, Pos. 62) - *“If she’s really very young and healthy, then I think that would rather argue in favor of managing it without antibiotics.”* (GP1, Pos. 28) - *“[The age] Doesn’t make that much of a difference for me. More from around, say, 60 or 70 years old - then rather yes.”* (GP12, Pos. 33) |
| **Risk of pyelonephritis:** The possibility of PN influenced some GPs, especially in patients with a history of PN, systemic signs of infection, or unclear clinical presentation. While some regarded PN as rare and of little concern, others had negative experiences and therefore considered it a decisive factor. Patients themselves occasionally expressed fear of PN. | - *“Yes, the expectation that this condition requires antibiotic treatment and that it won’t improve without antibiotics or even the fear that it could develop into a pyelonephritis. These fears are quite common among patients.”* (GP9, Pos. 54) - *“The fear of pyelonephritis - well, I don’t really encounter that in everyday practice as something one should truly be afraid of. The patients I see with pyelonephritis, they just have it right away, they’re healthy, and then suddenly really sick. They’re miserable, they have a high fever, a high CRP, they’re just not doing well...”* (GP6, Pos. 16) |
| **Antimicrobial resistance** Some GPs explicitly mention the topic AMR during the consultations, others do not. For many patients, AMR appeared rather abstract and less influential in decision-making. | - *“For the patients, no [the topic AMR] - they just want help, they want it [the symptoms] to go away, no matter how.”* (GP13, Pos. 58) - *“It’s [the topic AMR] less of an issue for patients, but increasingly so for me, I have to say.”* (GP14, Pos. 66) - *“Yes, for me that’s simply part of the discussion, whether to prescribe antibiotics or not, and I explain that there are more and more resistances and that it’s harmful for patients the more antibiotics they’ve had. Yes, absolutely, yes.”* (GP12, Pos. 49) |
| **Shared decision-making**: Decision-making was often described as a process between GPs and their patients. A trusting doctor–patient relationship, built through continuity of care, was seen as a key prerequisite. GPs reported routinely involving patients in discussions and asking for their agreement when suggesting non-antibiotic approaches. However, shared decision-making could be challenging in certain situations, especially when patients had strong expectations or limited understanding. | - *“Sometimes it’s a bit of convenience on the doctor’s side, and I include myself in that, because it’s simply easier, quicker, and less stressful to give out an antibiotic than to make a shared decision and explain pain management and anti-inflammatory therapy to the patient.”* (GP10, Pos. 3) - *“So I ask whether they would agree to first try a treatment with an anti-inflammatory medication, since we don’t always have to give an antibiotic right away. That’s not only the case with urinary tract infections, but with all infectious issues in general practice, we increasingly try other approaches first, for example with respiratory infections.”* (GP14, Pos. 22) - *“Yes, I don’t necessarily make that decision on my own. I make it together with the patient.”* (GP6, Pos. 18) |
| **Frequency of urinary tract infections:** A high frequency of UTIs generally prompted consideration of urological evaluation rather than directly influencing the decision to prescribe antibiotics. | - *“No, not really. I rather try to find out if there are any factors that might be contributing to it, for example, if it always happens after sexual intercourse. That’s where one could perhaps intervene and give some prophylactic recommendations to reduce the frequency.”* (GP1, Pos. 22) - *“No, in that case I would also first try to treat it without [antibiotics].”* (GP9, Pos. 42) |
| **Diagnostic testing:** Although GPs acknowledged that diagnostic testing (e.g., urine dipstick) is often unnecessary, it was frequently performed to support diagnosis, reassure patients, or justify a non-antibiotic approach. Negative results were sometimes used as a pedagogical tool to demonstrate the absence of infection and reinforce the decision against antibiotics. Conversely, visible hematuria was generally considered an indication for antibiotic therapy. | - *“If there are only moderate symptoms, just a few bacteria in the sediment, and the test strip is, let’s say, somewhat mixed, then I often say or suggest that we could initially do without an antibiotic.”* (GP1, Pos. 8) - *“If I have zero leukocyturia, zero, nitrite negative, and everything looks fine, then I tell the patients, I think you’re dealing with a different problem….”* (GP8, Pos. 32) |
| **Knowing the patient:** Familiarity with the patient strongly shaped GPs’ treatment decisions. Trusted and well-known patients were more likely to receive antibiotic-sparing management, as physicians felt confident in their judgment and follow-up. In contrast, for new or unknown patients, especially in walk-in or emergency settings, GPs tended to prescribe antibiotics more readily due to uncertainty about medical history and reliability of follow-up. | - *“Yes, when I know my patients, it’s much easier because I also know their medical history. In those cases, I can say, ‘Let’s take it easy, we’ll try conservative treatment and you just come back if it doesn’t get better.’ But when I see patients in the emergency service, I tend to prescribe antibiotics more readily because I don’t know how follow-up will work out. It’s also sometimes difficult to assess, some people are in a lot of pain and can be very demanding, which makes it challenging.”* (GP12, Pos. 21) |
| GP: General practitioner. Pos: Position (of the quote in the transcript). NSAID: Non-Steroidal Anti-Inflammatory Drug. PN: pyelonephritis. CRP: C-reactive protein. AMR: Antimicrobial resistance. SDM: Shared decision-making. UTI: Urinary tract infection. | |
