# Supplementary material for: General practitioners’ perspectives on treatment decisions and interprofessional care for women with uncomplicated urinary tract infections: An exploratory qualitative study
Source: PLoS One. 2026 Nov 11;21(11):e0362328. doi: 10.1371/journal.pone.0362328 (PMC13754150; doi:10.1371/journal.pone.0362328)
Supplement: Supplementary file 6 [file pone.0362328.s006.docx]

**Supplemental table S5:** Roles and activities of Medical Practice Assistants and Medical Practice Coordinators in the management of patients with suspected urinary tract infection

| **Phase of patient contact** | **Typical activities of MPAs/MPKs** | **Practice variation / influencing factors** |
| --- | --- | --- |
| Telephone contact | - First contact and point of triage for patients with urinary symptoms - Scheduling same- or next-day appointments or, if unavailable, referral to community pharmacies - Occasional counselling on fluid intake, use of phytotherapeutic agents, or analgesics | - Procedures rarely standardized - Extent of counselling depends on individual experience and confidence or has historically evolved - Some GPs are unaware of specific advice given at reception - No counselling in some practices due to time constraints, lack of reimbursement, legal uncertainty, or fear of missing serious conditions (e.g., pyelonephritis) |
| In-practice activities | - Performance of urine dipstick tests, occasionally microscopy, CRP or creatinine - In some cases, (brief) history-taking and screening for red flags - In high-demand settings, independent assessment and treatment suggestion (antibiotic or symptomatic), then approved by a physician | - Testing may be routine or conducted only on physician request - Level of autonomy varies with workload and experience |
| Post-consultation activities | - Additional counselling on medication use and supportive measures following the physician’s consultation | - Provided inconsistently across practices |
| CRP: C-reactive protein | | |
