# Supplementary material for: General practitioners’ perspectives on treatment decisions and interprofessional care for women with uncomplicated urinary tract infections: An exploratory qualitative study
Source: PLoS One. 2026 Nov 11;21(11):e0362328. doi: 10.1371/journal.pone.0362328 (PMC13754150; doi:10.1371/journal.pone.0362328)
Supplement: Supplementary file 7 [file pone.0362328.s007.docx]

| Supplemental table S7: Perceived advantages, barriers, and prerequisites for expanding MPA/MPK roles in uUTI management | | |
| --- | --- | --- |
| Aspects | Summary of findings | Quotes |
| Perceived advantages | - GPs described potential benefits of expanded roles, although their overall support and preferred degree of autonomy varied. - GPs emphasized that greater autonomy could enhance the professional image and attractiveness of the role, helping to counter workforce shortages and staff turnover. - Managing uncomplicated uUTIs was seen as a low-risk task suitable for well-trained MPAs. - Delegation was expected to reduce physicians’ workload, improve access to timely care, and allow greater focus on complex patients. - NPMS often have long-standing relationships and detailed knowledge of their patients. | - *“Yes, it also makes the profession more interesting. It’s a bit of a transitional job, especially for MPAs. They come, complete their training, and then three or four years later, they move on to something else.”* (GP4, Pos. 138) - *“The capacity of general practices would increase, the costs of care would decrease, and patients would be seen more quickly and get appointments faster. They would also feel more valued.”* (GP5, Pos. 108) - *“There’s a very simple reason: as a GP, I’m increasingly overloaded with trivial or minor cases, and I would gladly hand over tasks that other professionals can manage just as well. I actually welcome any support in that regard.”* (GP10, Pos. 79) - *“But at some point, we also have to change to some extent. I think if there are good new approaches, we should try them, and we can benefit from them, because we simply have too many patients, and we can no longer cover everything ourselves. In that sense, I believe there is also an opportunity here, especially if certain tasks could be delegated.”* (GP12, Pos. 83) - *“Well, I think the big opportunity is really the upgrading of the MPA role. That’s the greatest opportunity, that when you are an MPA, you’re no longer just the doctor’s assistant, but you can actually take on more responsibility yourself.”* (GP6, Pos. 88) |
| Perceived disadvantages and barriers | - Some GPs expressed scepticism, noting limited interest among MPAs and the time-intensive nature of training and supervision. - Concerns included variable competence levels, risk of misjudging patients, and the absence of standardized legal or financial frameworks. - Reimbursement and practice capacity were considered major constraints. - Several GPs also feared a “loss of simple cases,” reduced patient contact, and missed opportunities for preventive care. - Patient acceptance of being managed primarily by MPAs/MPKs was considered uncertain. | - *“It’s a direct contact, and you can quickly check whether any preventive measures are needed, how things stand with vaccinations, or how the patient is doing in general. I actually value these seemingly trivial, so-called ‘banal’ consultations very much.”* (GP6, Pos. 82) - *“There will be resistance, possibly from patients, because they might feel that their condition is not being taken seriously if they are not allowed to see a doctor but ‘only’ an APN or MPA/MPK.”* (GP10, Pos. 83) - *“It’s more that I try to keep the MPAs’ time on the phone short and to the point. Patients already complain that we’re hard to reach by phone, and if an MPA spends 8 or 12 minutes on a counselling call, that just doesn’t work. That happens only rarely. I don’t think any comparable practices in the area have more than two phone lines; we occasionally exchange ideas about how to handle the rush. I simply don’t have the financial resources to assign an additional MPA to phone counselling for such matters. It’s not feasible.”* (GP7, Pos. 48) - *“But the main problem is that it can’t be billed yet, right? It’s of no use to us - we can’t just provide care for free.”* (GP4, Pos. 90) - *“I have to say, I find it really refreshing, because in my practice I usually deal with topics that make my head spin. So it’s nice to occasionally have a small, straightforward topic again.”* (GP2, Pos. 77) |
| Prerequisites for expanding roles | - Adequate training, structured continuing education, and clearly defined legal frameworks were seen as essential. - Some GPs considered current training insufficient for independent clinical decision-making, particularly antibiotic prescribing. - Patient acceptance and economic feasibility (reimbursement and workflow efficiency) were identified as additional key requirements. - High commitment and willingness to engage in ongoing professional development are prerequisites for expanded responsibilities. - Transparent communication with patients about new professional roles and benefits | - *“I think, first, there would need to be specific training for MPAs on this topic, and second, there should also be accompanying media activity, in the sense that the new treatment guidelines clearly state that, for an uncomplicated bladder infection, the medical assistant can now make the initial decisions. This should also be communicated publicly, for example through the press.”* (GP14, Pos. 106) - *“As you already mentioned, the corresponding legal frameworks must be clearly defined, to ensure legal certainty for our MPAs, MPKs, and APNs.”* (GP10, Pos. 77) - *“And, well, patients would also have to perceive it as acceptable if they were ‘only’ seen by an MPA, so to speak. And the rules would have to be clear for everyone involved, and they would have to stick to them reliably, because it will only work if everyone follows the rules in the end.”* (GP1, Pos. 89) |
| GP: General practitioner, NPMS: non-physician medical staff, MPA: Medical Practice Assistants, MPK: Medical Practice Coordinators, APN: Advanced Practice Nurses, uUTI: uncomplicated urinary tract infections | | |
